# Predictability of Painful Stimulation Modulates the Somatosensory-Evoked Potential in the Rat

**DOI:** 10.1371/journal.pone.0061487

**Published:** 2013-04-16

**Authors:** Manon W. H. Schaap, Hugo van Oostrom, Arie Doornenbal, Annemarie M. Baars, Saskia S. Arndt, Ludo J. Hellebrekers

**Affiliations:** 1 Department of Clinical Sciences of Companion Animals, Faculty of Veterinary Medicine, Utrecht University, Utrecht, The Netherlands; 2 Rudolf Magnus Institute of Neuroscience, Utrecht, The Netherlands; 3 Department of Animals in Science and Society, Faculty of Veterinary Medicine, Utrecht University, Utrecht, The Netherlands; Université catholique de Louvain, Belgium

## Abstract

Somatosensory-evoked potentials (SEPs) are used in humans and animals to increase knowledge about nociception and pain. Since the SEP in humans increases when noxious stimuli are administered unpredictably, predictability potentially influences the SEP in animals as well. To assess the effect of predictability on the SEP in animals, classical fear conditioning was applied to compare SEPs between rats receiving SEP-evoking electrical stimuli either predictably or unpredictably. As in humans, the rat’s SEP increased when SEP-evoking stimuli were administered unpredictably. These data support the hypothesis that the predictability of noxious stimuli plays a distinctive role in the processing of these stimuli in animals. The influence of predictability should be considered when studying nociception and pain in animals. Additionally, this finding suggests that animals confronted with (un)predictable noxious stimuli can be used to investigate the mechanisms underlying the influence of predictability on central processing of noxious stimuli.

## Introduction

Rats are frequently used in the field of pain research [1], [2], and thus knowledge about nociceptive processing in those animals is of high importance when studying nociception and analgesic efficacy. Somatosensory-evoked potentials (SEPs) are used in humans and animals to increase knowledge about pain [3]. The SEP is a recording of the electroencephalogram (EEG), time-locked to a specific somatosensory stimulus. SEPs evoked by high intensity somatosensory stimuli are believed to reflect the processing of noxious stimuli [4], and correlate well with subjective pain ratings in humans [5], [6], adversity to pain in animals [7], [8] and are altered by anaesthetic and analgesic treatments in both humans [9] and animals [10], [11]. Therefore, the SEP can be considered a potential method to quantify acute pain. The SEP in animals is of special interest due to its potential to quantify and differentiate the sensory and affective component of acute pain [12].

During the recording of a SEP, other non-related processes are simultaneously going on in the brain, which have no direct relevance for the process of interest (i.e. processing of a noxious stimulus; [13]. These non-related processes are assumed to occur randomly across the EEG. Therefore, a representative SEP is obtained by averaging multiple recordings, by which non-noxious-related EEG activity is averaged out [13]. This procedure consequently necessitates repeated administration of the noxious stimuli. The way of administering these multiple stimuli (i.e. predictably or unpredictably) is a potential factor influencing the SEP in animals. In human research it has been found that unpredictable noxious stimuli lead to an increased SEP amplitude [14]. Although it is known that repeated exposure to unpredictable noxious stimuli results in long term negative emotional (i.e. depressive-like state) and physiological (i.e. stomach ulcers) effects in animals [15]–[17], the relationship between predictability of noxious stimuli and the animal SEP is yet unknown. However, influence of predictability on nociceptive processing may influence the results in animal studies that investigate (anti)nociception.

Predictability of a noxious stimulus can be manipulated by applying a classical fear conditioning paradigm. In this paradigm animals are exposed to neutral conditioned stimuli (CS, i.e. a tone) and to aversive unconditioned stimuli (US, i.e. an electrical pulse). If the CS is paired with the US, and the US becomes predictable, “conditioned fear” will develop as dominating emotion. However, if the CS and US are not paired, the US is unpredictable and temporally uncertain. Consequently, “anxiety” will develop as dominating emotion [18]. Fear is described as a phasic emergency reaction to an imminent threat, leading to physiological arousal, resulting in fight or flight when possible, or reducing the threat’s impact by, for example, decreasing pain sensitivity. Anxiety on the other hand, can be evoked by unpredictability of potential threats, and is characterized by a sustained state of increased vigilance and overall sensory sensitivity [18]. It is likely that these divergent effects of fear and anxiety on pain sensitivity [19] are reflected in the SEP in animals.

The influence of predictability of noxious stimuli on the SEP in the awake, freely moving rat was investigated using classical fear conditioning. On the premise that predictability of noxious stimuli plays a role in the processing of these stimuli in animals as in humans, we hypothesized that receiving shocks in an unpredictable fashion results in a higher SEP amplitude.

## Materials and Methods

### Ethical Note

The experimental protocol (DEC-DGK number: 2010.I.03.033) was peer-reviewed by the scientific committee of the Department of Animals in Science & Society, Utrecht University, The Netherlands, and approved by the Animal Experiments Committee of the Academic Biomedical Centre, Utrecht, The Netherlands. The Animal Experiments Committee based its decision on ‘De Wet op de Dierproeven’ (The Dutch ‘Experiments on Animals Act’, 1996) and on the ‘Dierproevenbesluit’ (the Dutch ‘animal experiments decree’, 1996). Both documents are available online at http://wetten.overheid.nl.

### Animals & Housing Conditions

Twenty-four adult male Wistar rats (HsdCpb:WU, Harlan Netherlands B.V., Zeist, The Netherlands), weighing 240–270 gram at the time of arrival were housed individually in clear 1500 U Eurostandard Type IV S cages, measuring 48×37.5×21 cm. Rats were provided with bedding material (Aspen chips), *ad lib* access to food (CRM, Expanded, Special Diets Services Witham, United Kingdom) and water and carton houses (Rat Corner House, Bio Services B.V., Uden, The Netherlands) as cage enrichment. The environment was temperature (21±2°C) and humidity (47±3%) controlled with an inversed 12∶12 h light-dark cycle (lights off from 7.00–19.00 hrs). A radio played constantly as background noise. All experimental procedures took place between 8.00–17.00 hrs. Animals were handled daily by the experimenters.

### Surgery

After an acclimatization period of three weeks, the animals underwent surgery for permanent implantation of epidural electrodes. Anaesthesia was induced in the rat’s home cage in a separate room under red light conditions, with 0.25 mg/kg fentanyl (i.p., Fentanyl Janssen®, Janssen-Cilag B.V., Tilburg, The Netherlands, 0.05 mg/ml fentanyl citrate) and 0.15 mg/kg dexmedetomidine (i.p., Dexdomitor®, Pfizer Animal Health B.V., Capelle a/d IJssel, The Netherlands, 0.5 mg/ml dexmedetomidine hydrochloride). After loss of the pedal reflex the animal was transported to the surgery room and, after endotracheal intubation, anaesthesia was maintained with isoflurane in 100% O_2_. The animals were provided with 8 ml of saline (s.c.) to support normal fluid balance and eye ointment to prevent drying of the cornea (Ophtosan® oogzalf, ASTfarma B.V., Oudewater, The Netherlands, 10000 IE vitamin A palmitate per gram). Subsequently, the animal was positioned in a stereotactic apparatus (model 963, Ultra Precise Small Animal Stereotaxic, David Kopf Instruments, Tujunga, CA, USA). Body temperature was monitored using a rectal probe thermometer and maintained at 37–38°C with an adjustable electrical heating mattress. In addition to clinical assessment (i.e. pedal reflexes), respiratory rate, heart rate, in- and expired CO_2_ and SpO_2_ were monitored continuously for assessment of anaesthetic depth and anaesthetic drug administration was adjusted appropriately. Following the skin incision but prior to detachment of the periostium from the neurocranium, 3 mg/kg lidocaine solution (Alfacaine 2% plus adrenaline, Alfasan B.V., Woerden, The Netherlands, 20 mg/ml lidocaine hydrochloride and 0.01 mg/ml adrenaline) was applied on the periostium. Five small wired stainless steel screws (tip diameter 0.6 mm, impedance 300–350 Ω, Fabory DIN 84A–A2, Borstlap B.V., Tilburg, The Netherlands) were implanted epidurally, according to stereotaxic coordinates as provided in the atlas Paxinos and Watson [20] above the vertex (Vx; 4.5 mm caudal to bregma, 1 mm right from midline), primary somatosensory cortex (S1; 2.5 mm caudal to bregma, 2.5 mm right from midline), anterior cingulate cortex (Acc; 1.5 mm rostral to bregma, 0.5 mm lateral from midline), and left and right frontal sinus (10 mm rostral to bregma, 1 mm lateral from midline). All electrodes were wired to an 8 pin receptacle (Mecap Preci-Dip 917-93-108-41-005, Preci-Dip Durtal SA, Delémont, Switzerland) and fixed to the skull with antibiotic bone cement (Simplex™ P bone cement with tobramycin, Stryker Nederland B.V., Waardenburg, The Netherlands, 0.5 g tobramycin per 20 g cement powder). The skin was closed in a single layer around the receptacle. Subsequently, anaesthesia was antagonized with 0.6 mg/kg atipamezole (i.p., Antisedan®, Pfizer Animal Health B.V., Capelle a/d IJssel, The Netherlands, 5 mg/ml atipamezole hydrochloride) and 0.05 mg/kg buprenorphine (i.p., Buprecare®, AST Farma B.V., Oudewater, The Netherlands, 0.3 mg/ml buprenorphine) in the animal’s home cage adjacent to the surgery room under red light conditions with extra oxygen supplied. After return of purposeful locomotion, the rat was transferred to the animals housing room.

Postoperative analgesia was provided with 0.05 mg/kg buprenorphine (s.c.) at 12 hour intervals for 3 days after surgery and 0.2 mg/kg of meloxicam (s.c., Metacam, Boehringer Ingelheim B.V., Alkmaar, The Netherlands, 5 mg/ml) at 24 hour intervals for 2 days after surgery. The weights of the animals were monitored daily until the pre-operative weight was reached, and allowed to recover for at least two weeks prior to the start of the first session.

### Stimulation and Recording Apparatus

Electrical stimuli were used as US and SEP-evoking stimuli, and administered as described earlier [7] to the epidermis of the left lateral part of the tail base, using a set of two bar electrodes (brass, diameter 2 mm), tapered towards the contact site and spaced at 3 mm from each other. The electrodes were fixed in a piece of plastic tube which enclosed the tail and was tightened by Velcro for maximal fixation. SEPs were elicited by multiple square-wave pulses of a 2 ms duration and stimulus intensity of 5 mA, generated with a Grass stimulator (Model S-88, Grass Medical Instruments, Quincy, Mass, USA), triggered by dedicated software written in house in a Labview environment (Labview 7.2, National Instruments Netherlands B.V., Woerden, The Netherlands). The stimuli were delivered to a Grass stimulation isolation unit and a constant current unit controlling the stimulus intensity. The number and frequency of electrical pulses differed per session and are described below. To prevent the animals from gnawing the cables, an Elizabethan neck collar, developed in house [21], was used during the administration of electrical stimuli in session 1, 2 and 3.

Sound stimuli consisting of a 10 s 2000 Hz tone, 75 dB sound pressure level generated by a sound generator (33120A, Arbitrary Waveform Generator, Hewlett Packard, Palo Alto, CA, USA), served as CS. Sound stimuli were presented by two speakers mounted in the covering lid of the box. The sound pressure level was verified by using a Modular Precision Sound Level Meter (type 2231, Brüel & Kjær, Nærum, Denmark).

For SEP measurements, the rat’s head mounted receptacle was connected to the recording device via a swivel connector (SLC-2, Plastics One, Roanoke, VA, USA). For each SEP recording, the accompanying ipsi-lateral frontal sinus electrode served as reference and the accompanying contra-lateral frontal sinus electrode served as signal ground. For each SEP, segments of 500 ms, 200 ms pre-, and 300 ms post-stimulus, were recorded and averaged online. All signals were amplified 1 million times, band-pass filtered between 3 and 300 Hz and digitized online at 10 kHz by data acquisition hardware (National Instruments, PCI-6251, Instruments Netherlands B.V., Woerden, The Netherlands). Additionally, a 50 Hz notch-filter was applied to eliminate interference from the power supply system. The SEPs measurements (session 1, 2 and 3) were carried out in a Plexiglas box measuring 40×28×30 cm with a stainless steel electrically grounded bottom, shielded by a Faraday cage. Different wallpapers were used to create different contexts between sessions. During session 1, the box was covered with wallpaper with alternating two cm vertical black and white stripes (context A). During session 2 and 3, the box was covered with plain white wallpaper (context B). The measurement of freezing behavior during session 4 was carried out in a different Plexiglas box (context C) measuring 39×29×32 cm, with white wall paper. Behavior was videotaped continuously throughout all sessions.

### Procedure

Prior to the surgery and four days after the surgery until the start of session 1, animals were habituated to wearing the Elizabethan neck collar in the animal housing room for 5 minutes daily. Two weeks after the surgery the measurements started, consisting of four sessions. All sessions were carried out in a separate experimental room, outside the room housing the animals. Before the start of each session’s measurements, animals were transported individually to the experimental room. Subsequently, animals were acclimatized in the Plexiglas box used for measurements for 20 minutes. Then, in all sessions except session 4, animals were fitted into the collar, the electrical stimulation device was fixed at the tail base and the head mounted receptacle was connected to the recording device. Experiments were performed in the “lights off” period under red light conditions.

**Figure 1:**
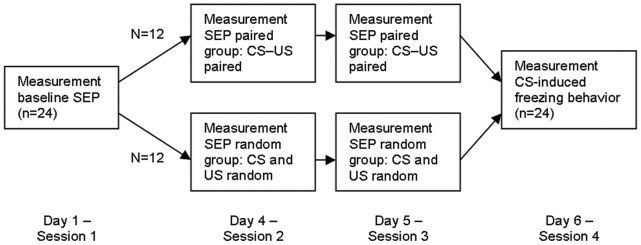
Schematic overview of the experimental procedure. See “procedure” for details.

**Figure 2:**
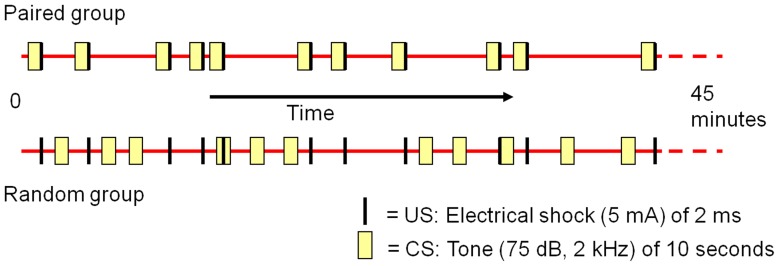
Schematic overview of the CS and US onsets in the paired and random group. The CS consisted of a 10 second tone. In the paired group, the US (an electrical pulse of 5 mA and 2 ms) always started 9 seconds after the CS onset, creating a temporal overlap between the CS and US in this group. In the random group, US onsets where identical to those of the paired group. The CS however, was presented randomly throughout the session. See “procedure” for details.

Figure 1 provides a schematic overview of the testing procedure. Animals were randomly assigned to either the paired (n = 12, in which the CS and US were paired) or the random group (n = 12, in which the CS and US were unpaired). In figure 2 the temporal difference (i.e. regarding contingency and contiguity) of the CS and US presentations between the paired and unpaired group is schematically represented. The experiment consisted of four sessions, including:

Session 1: A baseline-SEP was obtained in which both groups received 16 SEP-evoking stimuli with a constant frequency of 0.5 Hz.

Session 2: The paired group was subjected to a classical fear-conditioning paradigm, in which the US always started 9 seconds after the CS (figure 2). The interval between the CS-US pairings varied between 13 and 324 seconds (mean = 85 s; standard deviation = 88 s). In the random group, US onsets were identical to those of the paired group, but the CS was presented randomly throughout the session (figure 2). The intervals of the CS onsets were varying between 11 and 359 seconds (mean = 83 s; standard deviation = 93 s). Onset times were generated with the uniform random generator of the Analysis ToolPak in Microsoft Excel 2003. Both groups were exposed 32 times to the US and CS. SEPs (i.e. a single sweep) were recorded for each US presentation. The exact onset times of the CS and US in the paired and random group can be found in appendix S1.

Session 3: An exact repetition of session 2.

Session 4: Both groups were presented 5 times with the same CS as used in session 2 and 3. The CS-interval varied between 92 and 141 seconds (mean = 117 s; standard deviation = 23 s). The duration of freezing behavior (absence of all visible movements with the exception of breathing movements and pendulum motion of the head) was manually scored during every CS presentation [22].

### Data and Statistical Analysis

Calculations were performed with Microsoft Excel 2003 and statistical analyses were performed with SPSS 16.0. The data were statistically analysed using a mixed model regression. In this study, a mixed model regression is preferred over the commonly used ANOVA, as missing values do not result in list wise deletion of the animal and does not require imputation [23]. A backward strategy was adopted in which all non-significant interaction terms were removed. Grand mean centring was used (i.e. values are centred at 0) for all fixed factors, so the intercept could be interpreted and collinearity was prevented [23].

For the SEP-data the best fit was obtained by using the model with a random intercept. Fixed factors were recording site (Vx, S1 and Acc), group (paired, random) and session (1, 2 and 3) and their interactions. P-values ≤0.017 (Bonferroni correction for the number of dependent variables, namely 3) were considered significant. In case of significant group*session interaction simple effects were tested in a Bonferroni corrected post-hoc test using a nested model, estimating the effect of session 2 and 3 compared to session 1 (baseline) per group. Dependent variables were the amplitudes of positive-to-negative components, including P1-N1, N2-P2 and P3-N3 (see figure 2). The latencies of these peaks are listed per recording site in table 1. The Q-Q plots of the residuals indicated a normal distribution for all variables.

**Table 1:** Latencies in milliseconds (± SEM) of each peak per recording site.

	Vx	S1	Acc
P1	13.7 (±0.1)	13.4 (±0.1)	14.6 (±0.2)
N1	18.5 (±0.1)	18.0 (±0.1)	18.1 (±0.2)
N2	32.8 (±0.4)	36.1 (±0.5)	43,8 (±0.6)
P2	56.0 (±0.5)	57.9 (±0.6)	59.5 (±0.5)
P3	93.2 (±0.9)	98.0 (±0.9)	105.0 (±0.8)
N3	138.5 (±1.1)	142.2 (±1.2)	145.6 (±1.1)

Electrodes were implanted epidurally above the vertex (Vx), primary somatosensory cortex (S1) and the anterior cingulate cortex (Acc).

The scorings of freezing behavior by the experimenter [MS] and by the second observer who was unaware of the aims and procedures of the experiment correlated highly (Pearson’s r = 0.83, n = 45, p<0.001). The best fit was obtained by using the model with a random intercept. Fixed factors were group (paired, random) and CS presentation (1 through 5) and their interaction. The Q-Q plot of the residuals indicated a normal distribution for all variables.

## Results

### Missing Values

For the Vx-SEP, no missing values occurred. Technical problems occurred in the S1-SEP of four rats during the first session, the Acc-SEP of three rats throughout all sessions, and S1-SEP of two rats throughout all sessions. In total, 72 (Vx-SEP, n = 36 for both paired and random), 62 (S1-SEP, n = 31 for both paired and random) and 63 (Acc-SEP, paired: n = 33 and random: n = 30) SEPs were analysed.

### Somatosensory Evoked Potentials

**Figure 3:**
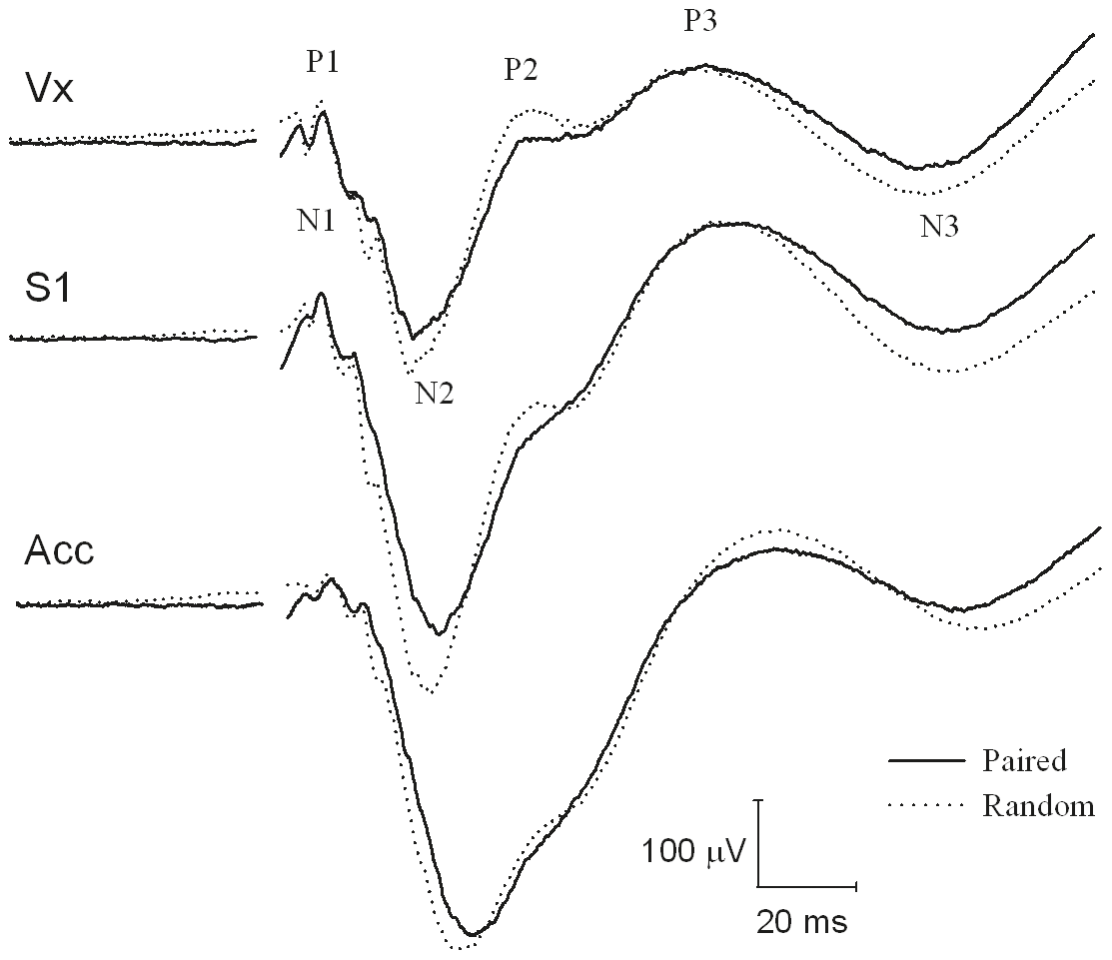
The grand means of the SEP for the different recording sites. Electrodes were implanted epidurally above the vertex (Vx), primary somatosensory cortex (S1) and the anterior cingulate cortex (Acc). The average of the SEP signals obtained during session 2 and 3 for the paired (n = 12) and random group (n = 12). The curve interruption denotes the stimulus onset.

**Figure 4:**
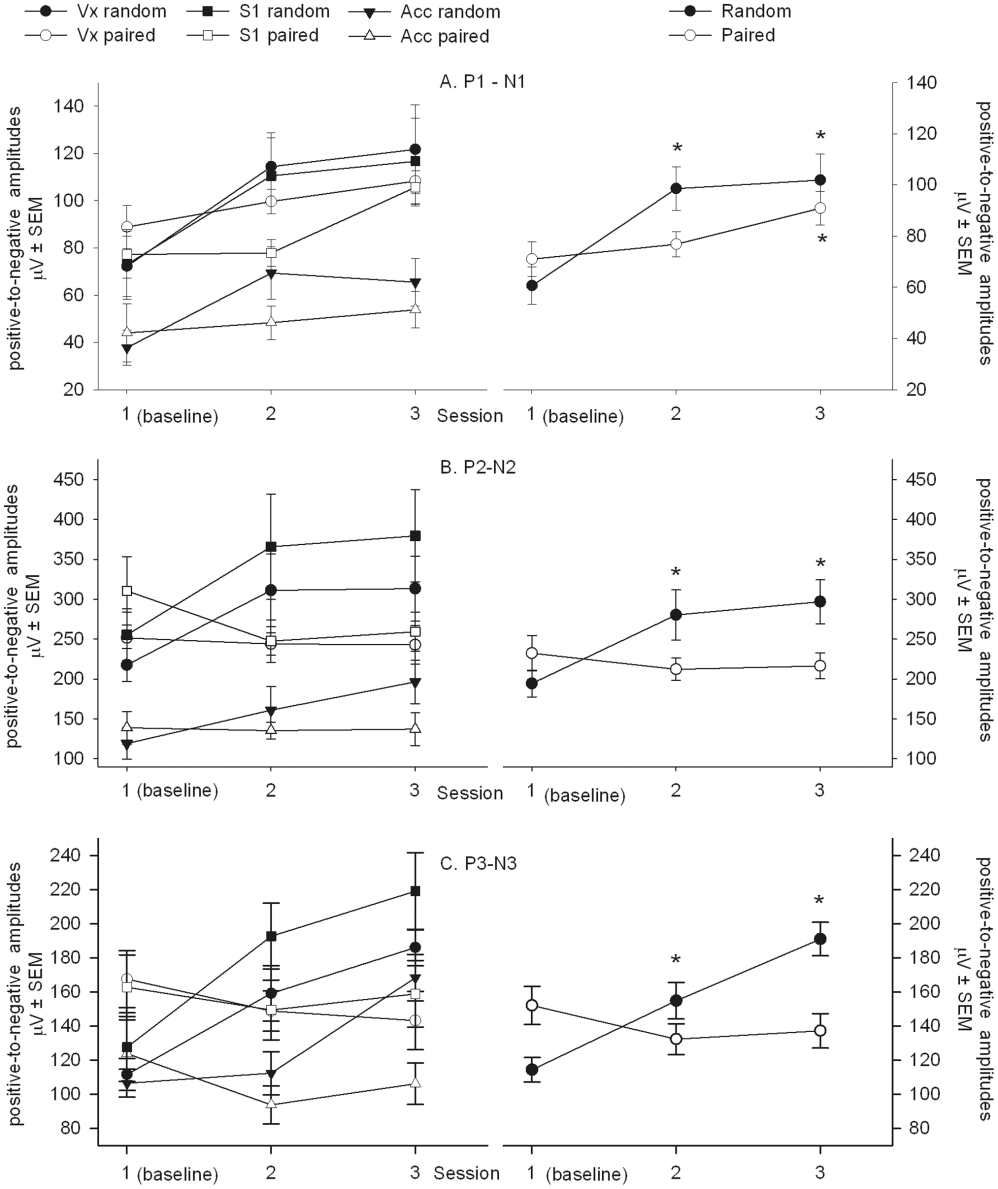
SEP signals during different conditions. Electrodes were implanted epidurally at different recording sites above the vertex (Vx), primary somatosensory cortex (S1) and the anterior cingulate cortex (Acc). The mean positive-to-negative amplitudes per session are shown by group and recording site (left column) and by group (right column) for the (A) P1-N1, (B) P2-N2 and (C) P3-N3. Data are represented as mean µV ± SEM. See “results” for details. *p<.01 versus session 1 (baseline) amplitude.

The average SEP waveforms of the paired and random group during session 2 and 3 with its peak definitions are shown in figure 3. The P1-N1 amplitude showed no group*session*recording site, group*recording site or session*recording site interaction (F_4,173.57_ = 0.27, p = 0.90, F_2,175.54_ = 0.39, p = 0.67 and F_4,173.58_ = 1.02, p = 0.40, respectively). A group*session interaction was found (F_2,173.57_ = 6.21, p<0.01). Post-hoc analysis showed that the amplitude increased compared to baseline in the random group during session 2 and 3: t_173.61_ = 5.85, p<0.001 and t_173.61_ = 6.37, p<0.001, respectively, whereas the amplitude of the paired group did not differ from baseline during session 2: t_173.62_ = 0.89, p = 0.38 but was increased during session 3: t_173.62_ = 3.03, p<0.01 (figure 4A).

The P2-N2 amplitude showed no group*session*recording site, group*recording site or session*recording site interaction (F_4,173.21_ = 0.63, p = 0.64, F_2,175.51_ = 1.60, p = 0.21 and F_4,173.21_ = 0.27, p = 0.90, respectively). A group*session interaction was found (F_2,173.22_ = 9.44, p<0.001). Post-hoc analysis showed that the amplitude increased compared to baseline in the random group during session 2 and 3: t_173.26_ = 3.79, p<0.001 and t_173.27_ = 4.63, p<0.001, respectively, whereas the amplitude of the paired group did not differ from baseline during session 2 and 3: t_173.28_ = −1.23, p = 0.22 and t_173.28_ = −1.03, p = 3.05, respectively (figure 4B).

The P3–N3 amplitude showed no interactions between group*session*recording site, group*recording site, or session*recording site (F_4,173.31_ = 0.43, p = 0.79, F_2,176.89_ = 1.38, p = 0.26 and F_4,173.31_ = 1.07, p = 0.37, respectively). A group*session interaction was found (F_2,173.22_ = 19.20, p>0.001). Post-hoc analysis showed that the amplitude increased compared to baseline in the random group during session 2 and 3: t_173.32_ = 3.59, p<0.001 and t_173.32_ = 6.99, p<0.001, respectively, whereas the amplitude of the paired group did not differ from baseline during session 2 and 3: t_173.33_ = −2.14, p = 0.03 and t_173.33_ = −1.69, p = 0.09, respectively (figure 4C).

### Freezing Behavior

**Figure 5:**
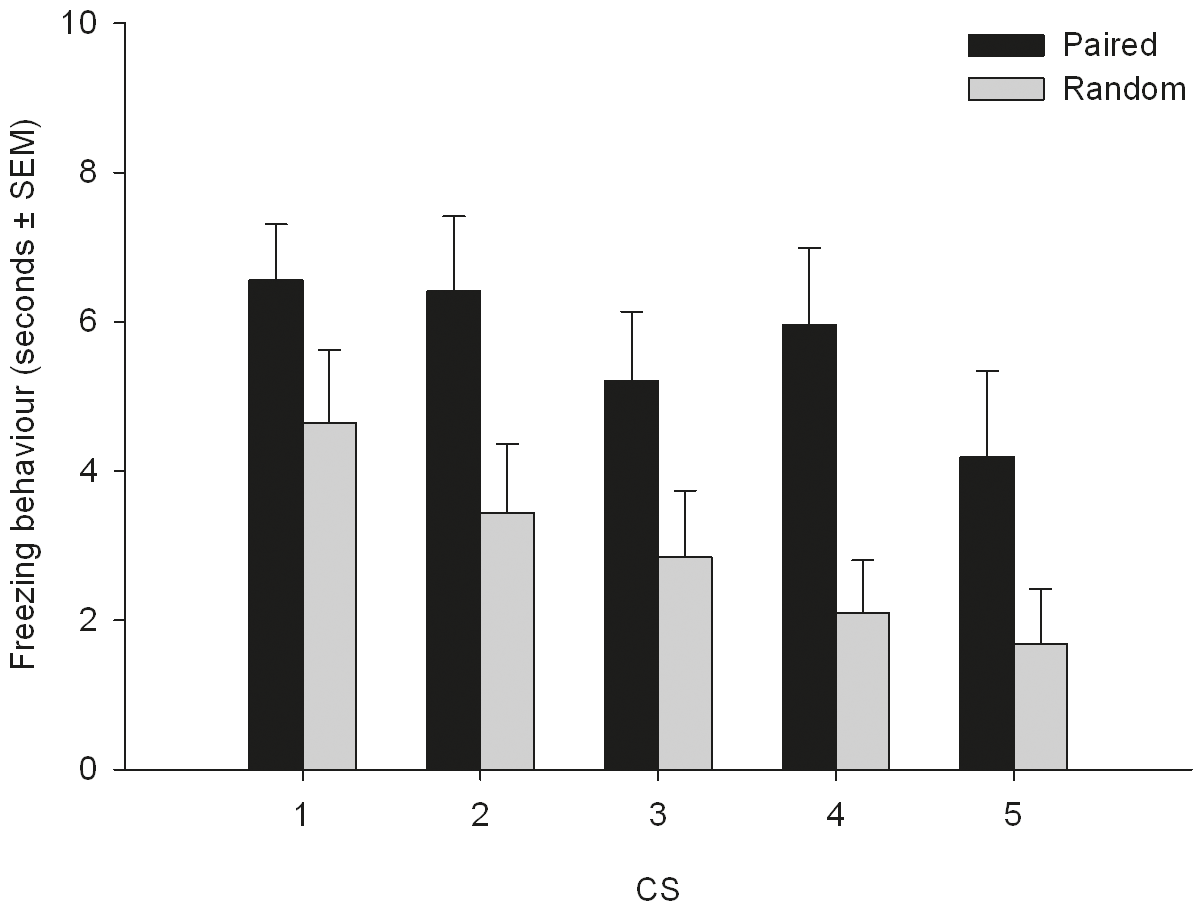
Freezing behavior during CS presentations of session 4. The paired group showed more freezing behavior than the random group. Freezing behavior decreased over time. See “results” for details. Data are represented as mean seconds ± SEM.

The CS induced freezing behavior during session 4 is depicted in figure 5. No interaction between CS presentation and group was found (F_4,95.15_ = 0.47, p = 0.76). The paired group showed more freezing behavior (5.7 s ±0.44) than the random group (2.9 s ±0.39; F_1,24_ = 11.13, p<0.01) and freezing behavior decreased over CS presentations in both groups (F_4,95.14_ = 3.49, p = 0.01).

## Discussion

In both human and animal studies of pain and (anti)nociception SEPs are frequently used to study the neural processing of noxious stimuli and the effect of antinociceptive treatments [3]. The generation of a SEP requires repeated administration of the noxious stimuli [13]. These multiple stimuli can either be administered in a predictable or unpredictable fashion. The principal finding of this study is that the SEP in rats, when compared to baseline, increased when SEP-evoking stimuli were unpredictable (random group), whereas no increase was observed when these stimuli were predictable (paired group). Therefore, it is concluded that predictability of noxious stimuli plays an important role in the processing of these stimuli in rats, as is shown to the case in humans [14].

Predictability was manipulated by applying classical fear conditioning. As the paired group showed more CS-induced freezing behavior during session 4 than the random group, it can be concluded that a strong association between the CS and the US was present during sessions 2 and 3 in the paired group. Therefore, it is assumed that noxious stimuli (i.e. SEP-evoking stimuli) during sessions 2 and 3 for the paired group were predictable, and for the random group unpredictable, and thus the animals from the paired group (but not from the random group) could predict the US onsets. As the only differences between groups were the contingency and contiguity between CS and US (and hence the predictability of the US; [24], differences shown in the SEP signal between groups are attributable to differences in predictability of the US.

Human studies showed that unpredictable noxious stimuli result in increased anxiety and pain sensitivity [14], [25]. The increase occurred concurrently with the increased SEP [14], [26]. Negative emotional effects of unpredictable noxious stimuli have been hypothesized to occur in animals as well [15]–[17] and it is likely that anxiety developed as a dominating emotion in the random group [18], [27]. Our results support the hypothesis that emotions such as anxiety play a role in the processing of nociceptive stimuli in animals, although the exact role in the generation of the increased SEP in animals needs to be addressed in future studies.

Our findings show that amplitudes of SEPs evoked by unpredictable stimuli are increased compared to baseline, this being in direct contrast to the amplitudes of SEPs evoked by predictable stimuli. The finding that the SEP amplitude in the paired group does not change, while the SEP amplitude of the unpaired group increases with respect to the baseline measurement, seems counterintuitive if one would assume that the stimuli during the baseline recordings were unpredictable. However, during baseline recordings a train of 16 stimuli with a fixed interstimulus interval (ISI; the temporal interval between the offset of one stimulus to the onset of another) was used, as such making these stimuli predictable (13, 23). Consequently, the present findings do support our hypothesis that unpredictable stimuli lead to higher SEP amplitudes than predictable stimuli. The paired group receives stimuli that are predictable during baseline and during sessions 2 and 3, resulting in the SEP amplitude remaining similar to baseline. In the unpaired group, stimuli during baseline were predictable and unpredictable during session 2 and 3, explaining the increase in SEP amplitude during session 2 and 3 with respect to baseline. In conclusion, the present results of change in SEP amplitude adequately reflect the changes in predictability of the stimuli over sessions and between groups.

Although multiple studies showed that predictability of noxious stimuli affects brain activity in response to nociceptive stimuli, the strength of these effects may differ depending on the measurement technique used. A human functional magnetic resonance imaging (fMRI) study showed an increased blood-oxygen-level dependence (BOLD) signal in brain areas associated with the sensory component of pain (i.e. primary somatosensory cortex (S1)) when shocks were predictable. When shocks were unpredictable, brain areas associated with the affective component of pain (i.e. the anterior cingulate cortex (Acc)) showed a higher BOLD signal [25]. Using single cell recording, Wang et al. (2008) showed that rat’s laser-evoked neuronal activity (i.e. unit responses) was increased in the Acc and S1 when the noxious laser stimuli were predictable [28]. Although results of different measurement techniques (i.e. fMRI, EEG and single cell recording) do not necessarily contradict each other and results of one measurement technique are not preferred over the other, this discrepancy highlights the importance of taking the technique used into account when interpreting and comparing results of different studies. The SEP is the only neurophysiological technique used in both animals and humans investigating the effect of predictability of noxious stimuli. It is a valuable technique in both fundamental and translational research that can be recorded both in awake animals as well as in humans, as opposed to fMRI (were the animal has to be anesthetized) and single cell recording (which is considered unethical in humans under normal circumstances). When comparing the effect of predictability of noxious stimuli on the SEP, animals and humans show similar effects of predictability on neural processing.

Importantly, electrical stimulation does not specifically affect nociceptive fibers only [29], therefore the SEP (as measured in this study) next to consisting of nociception related responses may also relates to non-nociceptive components. However, the fact that the SEP is modulated by the u-opiate receptor agonist fentanyl [12], which specifically modulates nociceptive processing [30], [31], the strong correlation between freezing behaviour and the SEP [8], and the frequently observed aversive behaviours following stimulation in this study (i.e. jumping and vocalization; unpublished data) together are in strong support for a noxious component. However, even when using stimulation types specifically activating nociceptors, in the literature to date it is furthermore hypothesized that the SEP, rather than specifically representing the processing of nociception and/or perception of pain, may primarily be representative for a more general defensive mechanism which plays an important (but non-specific) role in pain [32]. Specifically, the cortically-derived SEP represents processes involved in detecting, focussing attention and subsequently selecting appropriate responses to potentially significant (i.e. threatening or salient) stimuli, regardless of the stimuli’s modality. According to this view, the SEP provides an indirect rather than direct representation of pain perception per se [33], [34]. However, as these non-specific processes (such as affective salience or unpleasantness) are an intrinsic part of pain experienced, the SEP remains a valuable method to investigate the functioning of the nociceptive system.

One factor potentially confounding the current results is that in the paired group the US in time always overlapped with the CS, resulting in simultaneous processing of the CS and US in this group (but not in the random group). However, in the current study no differences were found between the baseline (where only a US but no CS was present) and session 2 and 3 in the paired group (where both the US and CS were present). If the simultaneous processing of the CS and US alters the processing of the US (and thus the SEP), session 2 and 3 would have differed from the baseline in the paired group. Furthermore, Oka et al. (2010) showed that unpredictability increased the amplitude of SEPs in humans when predictability was manipulated by a combination of variations in ISIs and stimulus intensities. A fixed ISI and fixed intensity sequences creates maximal predictability and variable ISIs and random intensities create maximal unpredictability [14]. Therefore, it can be hypothesized that the SEP’s increased amplitude following unpredictable noxious stimuli are not caused by the mode by which noxious stimuli are predicted, but by predictability itself. This effect of predictability may not be specific for nociceptive stimuli and affect the processing of other stimuli as well.

When creating (un)predictability by using fixed or random ISIs (as in [14], [25], it should be considered that factors other than temporal unpredictability might play a role in the altered neural activity. Repetition suppression (RS) is found to be a robust phenomenon, which leads to a reduction of cortical activity after repeated stimulation, especially in case of short ISIs (i.e. <2 seconds) [35]. Interestingly, in the case of noxious stimuli and the application of short ISIs, this reduced cortical activity is not accompanied with an altered pain perception [36]. Several mechanisms are proposed to explain this RS at short ISIs [37], including refractoriness in nociceptive afferent pathways [38] and temporal predictability [36]. However, since in the current study ISIs of the US (i.e. the SEP-evoking stimuli) were identical between the paired and random group and furthermore relatively long (i.e. 2 seconds during the baseline measurement and varying from 13 to 324 seconds during session 2 and 3). Therefore, in our view, RS can be excluded here as an influencing factor with respect to the interpretation of the results.

It has been shown that attention to a noxious stimulus affects pain sensitivity [39] and increases the SEP’s amplitude in humans [40]. In the current study none of the groups was experimentally distracted from the SEP-evoking stimuli, making it unlikely that the differences in the SEP’s amplitude can be explained by the presence or absence of attentional focus on the SEP-evoking stimuli. It is deemed highly probable that in both groups, the attention was directed to the SEP-evoking stimuli through bottom-up selection [32], i.e. the involuntary attraction of attention to salient stimuli (i.e. stimuli which stand out relative to other stimuli). However, in contrast to the paired group, the random group was exposed to stimuli which were temporally unpredictable, thus inducing a difference in top-down attention (i.e. cognitive expectations; [36]. Importantly, both groups are likely to differ in their emotional state (i.e. fear versus anxiety), and inherently the type of attentional focus will differ. Receiving noxious stimuli in an unpredictable fashion (random group) causes a state of anxiety associated with sustained attention, whereas predictable stimuli (paired group) cause a state of fear associated with selective attention (for a comprehensive review on this topic see reference [18]. Therefore, we hypothesize that our experimental design allows for a distinction between anxiety (created by unpredictability in the random group) and fear (created by predictability in the paired group), consequently associated with different effects on pain sensitivity [19], [25], [27]. The exact role of anxiety and fear in the generation of the SEP however, remains elusive and needs to be addressed in future studies.

### Conclusions

This study shows that the predictability of noxious stimuli is an important factor affecting the SEP in animals, as is shown to be the case in humans. Therefore, when studying the neural processing of a noxious stimulus using the SEP, predictability of the noxious stimuli should be considered when designing and interpreting results of animal experiments. As it is likely that this influence is not restricted to EEG derived parameters, this potential effect should be considered in animal models of pain in general. Additionally, this finding suggests that animals confronted with (un)predictable noxious stimuli can be used to investigate the mechanisms underlying the influence of predictability on central processing of noxious stimuli.

## Supporting Information

Appendix S1 **The exact onset times of the CS and US in the paired and random group during session 2 and 3.** The CS consisted of a 10 second tone. In the paired group, the US (an electrical pulse of 5 mA and 2 ms) always started 9 seconds after the CS onset, creating a temporal overlap between the CS and US in this group. In the random group, US onsets where identical to those of the paired group. The CS however, was presented randomly throughout the session. See “procedure” for details.
(DOCX)
