# Supplementary material for: Predictability of Painful Stimulation Modulates the Somatosensory-Evoked Potential in the Rat
Source: PLoS One. 2013 Apr 16;8(4):e61487. doi: 10.1371/journal.pone.0061487 (PMC3629015; doi:10.1371/journal.pone.0061487)
Supplement: Appendix S1 — The exact onset times of the CS and US in the paired and random group during session 2 and 3. The CS consisted of a 10 second tone. In the paired group, the US (an electrical pulse of 5 mA and 2 ms) always started 9 seconds after the CS onset, creating a temporal overlap between the CS and US in this group. In the random group, US onsets where identical to those of the paired group. The CS however, was presented randomly throughout the session. See “procedure” for details. (DOCX) [file pone.0061487.s001.docx]

Appendix S1

| **Number** | **Paired group: Onsets CS (in seconds)** | **Paired and random group: Onsets US (in seconds)** | **Random group: Onsets CS (in seconds)** |
| --- | --- | --- | --- |
| 1 | 17 | 26 | 89 |
| 2 | 53 | 62 | 198 |
| 3 | 68 | 77 | 219 |
| 4 | 232 | 241 | 437 |
| 5 | 325 | 334 | 467 |
| 6 | 396 | 405 | 485 |
| 7 | 424 | 433 | 529 |
| 8 | 542 | 551 | 545 |
| 9 | 556 | 565 | 609 |
| 10 | 573 | 582 | 702 |
| 11 | 830 | 839 | 728 |
| 12 | 891 | 900 | 772 |
| 13 | 935 | 944 | 865 |
| 14 | 955 | 964 | 911 |
| 15 | 978 | 987 | 961 |
| 16 | 1263 | 1272 | 1052 |
| 17 | 1333 | 1342 | 1064 |
| 18 | 1346 | 1355 | 1084 |
| 19 | 1463 | 1472 | 1111 |
| 20 | 1508 | 1517 | 1143 |
| 21 | 1533 | 1542 | 1154 |
| 22 | 1549 | 1558 | 1172 |
| 23 | 1873 | 1882 | 1269 |
| 24 | 1886 | 1895 | 1289 |
| 25 | 2076 | 2085 | 1318 |
| 26 | 2106 | 2115 | 1377 |
| 27 | 2229 | 2238 | 1588 |
| 28 | 2265 | 2274 | 1947 |
| 29 | 2297 | 2306 | 2023 |
| 30 | 2378 | 2387 | 2319 |
| 31 | 2609 | 2618 | 2342 |
| 32 | 2656 | 2665 | 2657 |
